# Hospitals and Sick Children

**Published:** 1890-05-31

**Authors:** 


### THE STORY OF THE HOSPITALS.

### HOSPITALS AND SICK CHILDREN.

"A Little Child Shall Lead Them."

### Chapter I.—Introductory.

#### AN EAST-END BABY.

"The baby—the baby that dropped from the skies."

So sang the Board School children, languidly enough, one February afternoon; but when at last school-hours were over, and little Esther Hawkins climbed the steep dirty staircase that led to her home—a single room in a teeming house situated in a teeming East-end court—she found that the song had been prophetic, and that a baby had indeed come into that house, as several babies had done before—whether from the skies or not she did not know. It had chosen its time better than might have been expected of so young a thing; for in that room ate and slept eight souls, the father and mother, three little ones, two big boys, and a girl of seventeen—these last three being Hawkins' children by a former marriage; and had the big, wistful eyes opened upon such a party, as they very well might have done, it would have been less pleasant for the mother, if not for the babe.

#### A HARD LOT.

Such a poor little thing as it was, too—tiny, shrunken, and weak, as indeed was little wonder, for food was always short in that house when work was slack at the docks—aye, and sometimes when it was not; and the mother, a shiftless, untidy, but really good-hearted and affectionate soul, had had a hard, anxious time of it of late. She had an industry of her own, sack-making, and had worked at that until the last moment, standing all the time, that she might push the needle into the stiff canvas with more force. Moreover, she had had a terrible fright, her husband having one day come home maddened by some specially vile decoction of the publican's, and attacked little Esther and herself with the poker, the teapot, anything that came to hand. However, the baby was born alive, as she had hardly expected it would be; and in spite of all troubles, past and prospective, the sight of that tiny face at her breast, with its big, uplifted eyes—eyes which seemed to grow more wistful and pathetic day by day—comforted her heart, and led her thoughts away from the sordid cares and anxieties which had hitherto filled up the horizon of her life.

Whether or no it can be said of all our nineteenth-century babies, that "Trailing clouds of glory do they come, from God, who is our home," it certainly seemed that with this one "heaven lay about it in its infancy;" and no doubt it was with some instinctive feeling of this kind that the mother would hear of none but a Scripture name for her infant—Ebenezer.

#### A WEST-END BABY.

On the same day, and at very much the same time, there was a birth in a fashionable West-end house—the home of a young barrister, Julian Heywood, and his wife. They were happy in the possession of each other's love, besides having every luxury that wealth could procure; and the one bitter drop in the cup of their bliss had been but this, that for eight years the child for which both parents eagerly longed had delayed its coming. Now at last it was here; and when the eager young father crept on tiptoe into the half-darkened, sweet-smelling room, and softly drawing back the pink and white curtains, bent down over the two heads that lay so close together, the sweet flower-like face of the mother flushing a delicate pink with the wonder and the joy of it, and the tiny, downy oval—for all the world like a magnified bud of the willow-palm—he never spoke a word beyond that first exclamation, "My darlings!"—but stood lost in dreams of the days to come, when a little son should trot by his side along the streets or the country lanes, when a bigger son should visit with him the old home of his childhood, should stand with him beneath the grand grey walls of his Alma Mater, and he himself should live his childhood and his youth over again in that bright young presence.

"See, Julian, how sweet and good he is! He never cries, though I've been trying to wake him all this time to let you see his eyes—the little lamb!" said the mother's voice; and Julian woke from his reverie, and laughed.

"A lamb? No, no! My boy is to be a lion, not a lamb," he said.

And "Lionel" the child was called.

### Chapter II.—A Catastrophe.

A SILENT COMFORTER.

Five years passed away; and widely different though the lives of the two little fellows were, both alike, in humble and unconscious imitation of the Master whose cross was on their foreheads, grew "in favour with God and man." With regard to stature, indeed, little Eben would never accomplish much; he was small and wasted-looking, and could never walk far without leaning against some wall or railing, or dropping without more ado to the ground, as though wearied out. And before he had grown out of babyhood, the unwelcome truth forced itself upon his mother—her child would be deaf and dumb. Moreover, there was a mysterious cloud over his mind, of which she knew nothing as yet, putting down all his unlikeness to her other children to the fact of his being deaf. You could not call him an idiot; there was none of that mopping and mowing which is so distressing and almost revolting to witness; it was rather as though he were a little removed from the world—as though a thin, impalpable veil were hung between him and it. Was it that the clouds of glory were still trailing round him? However that might be, his presence brightened and blessed that squalid, poverty-stricken home. His placid, peaceful little face was always the first the children looked for when they returned from school; the sound of his bitter crying would often stop what bade fair to be a protracted quarrel among his bigger brothers and sisters; his tender little caresses soothed the pain at the mother's heart, when work was short, or the eagerly-reckoned-on money melted away at the public-house before she could even get a sight of it.

A LION AND A LAMB.

As for little Lionel, to say that he was the sunshine of his parents' life is to put it mildly; to them it seemed as though he were that very life itself. Nothing Julian did but had some relation, direct or indirect, to him; and while the father's heart delighted in the sturdy limbs, the glorious health, and the bold, manly ways of his little son, the mother rejoiced especially over his sunshiny curls, his clinging affection, and the tender-heartedness which early made itself visible.

"We were both right; he is a lion, and a lamb, too!" she would sometimes say; and so it really was.

His powers of observation, also, were very early developed; and when he was only two, and his mother was carrying him upstairs, his little eyes were quick to discern the signs of fatigue on her face. "Lion's too heavy; Marmee mustn't cally him," he lisped.

A BABY PHILANTHROPIST.

His parents were so wrapped up in their happiness that they gradually withdrew into a little world of their own, only coming into contact with other people where it was absolutely necessary; but little Lion's interest in the world around him was keen and sympathetic, and to his nurse he would talk by the hour of the poor little children who, as she told him, had no nice clothes to wear, or food to eat, such as he had. "Give that poor little boy Lion's dinner—oh Nursie, do!" he would implore, when a ragged little urchin passed his nursery window; and when once a little girl with a wooden leg went by, he broke into bitter sobs, telling his mother that he had seen a little girl who was nearly dead,—all the flesh and skin were coming off her bones.

The mother loved her little Lion for his tender-hearted sympathy; but so absorbed was she in him that it did not lead her, as it might have done, to ponder the lot of the numberless children needing the pity and help which she and her little son, rich in health and comforts, might so easily bestow.

LIONEL'S WHIM.

Five years passed away, as we have said; and the two children met—for one brief moment. Lionel's birthday was always kept as a grand occasion; the whole day was given up to him, and he was allowed to choose in what way he would spend it. This year he was to have a Christmas-tree, and a Punch and Judy show; but for the afternoon, nothing would content him but that his father should take him to the East-end, in which his nurse had lived as a child, and about which she was always talking to him. "Let me see the big river and the ships, father," he urged, "and the funny sailors walking about, and the poor little children playing shuttledore in the road—they never play in our square." And the father, though he thought it an odd whim of the child's, consented to please him.

AN EAST-END RAMBLE.

Lion was a capital walker for a boy of his years, and enjoyed himself thoroughly, from the time when they stepped from the underground railway into the busy East-end street, till, after rambling about the docks, and peering down numberless little streets and alleys, they found themselves once more in the broad thoroughfare, their East-end experiences nearly over. The twilight was falling, and Lion felt tired, and as though he wanted his tea; but when his father pulled up to speak to a friend who suddenly slapped him on the shoulder, the little boy stood contentedly by his side, taking in, in his wise little way, all that there was to be seen. I think his anxiety to see the "poor little children" in their natural haunts had been caused by a fear that nurse was not wrong after all, and that they were all shivering and wretched, like the little beggars whom he had watched from his window. Now he had seen merry faces, and heard playful shouts, in the midst of rags and squalor, and his little mind was relieved.

EBEN APPEARS ON THE SCENE.

But as he stood there by his father's side, he beheld a dismal figure emerge from an alley not many yards away—a tiny little fellow in ragged clothing, with a white, pinched face, and big, wistful eyes, from which the tears were slowly falling. For a minute or two the children stood looking at each other, Eben in his rags and dirt, and the healthy, finely-made little gentleman, with his bright eyes and rosy cheeks, and the sunshiny curls falling upon his dark-blue velvet suit. But while Lion was all attention and eager sympathy in a moment, his wide-open eyes taking in every detail of the ragged little figure before him, Eben's interest in him was only momentary. The wistful eyes wandered away from the bright little stranger to where across the road the lights of a gin-palace had already begun to flare. There was a minute or two of timid indecision, of frightened observance of the crowd of drays and 'busses that loomed vague but terrible in the twilight; then the decision was made, and little Eben, with a sudden start, made for the gin-palace.

"LION'LL HELP YOU!"

What a moment for Lion! Now at last that divine longing to help and to save that had burned so ardently within his little soul could find expression. He saw Eben's sudden plunge, and the manifest terror with which he dodged, and just escaped, the heavy forefeet of a great dray-horse; and in a moment he had snatched his hand from his father's and sprung to Eben's side, with a shrill, clear little cry—"Lion's coming! Lion'll help you!"

The spirit truly was willing, but the flesh was weak, so far, at least, as the success of his endeavour was concerned. There was a moment of horrible suspense for the startled father; and then the two little figures, now hand in hand, shot staggeringly forward, struck by the shaft of a passing cart, and disappeared from view, amid cries and shouts, and a general scuffling of hoofs, as one horse after another was pulled up on its haunches, and came to a standstill.

"OF SUCH IS THE KINGDOM OF HEAVEN."

Only a street accident. Scores of such happen every week in the busy streets of our metropolis; and in a minute more the ceaseless hum had begun again, and the spectators had gone on their way, thinking each of his business or pleasure, the work he had left, or the home he was going to. But upon Julian Heywood's life the blackness of darkness had descended; all joy, all hope, seemed to leave it for ever as he gazed at the mangled form of his little son, and heard those staggering words, "He is dying!"

"To the hospital!" many voices had cried, as the two children were picked up; but the friend to whom Julian had been talking shook his head. He was the vicar of the parish, a man of much experience and some knowledge of surgery; and a brief examination of Lion convinced him that the child had but a few moments to live.

"Better wait a minute," he said. "Yes; the other can go—I doubt if he is seriously hurt; but your child, Heywood—— Here, bring him into this shop; they'll take him in gladly. There's a doctor living close by, and we can hear what he says about moving him."

But it was as he thought; Lion had not five minutes to live. He drew but a few long breaths, opened his eyes, and fixed them gravely, wonderingly, on his father, and then he died.

An hour later, when the poor mother opened the door of that little confectioner's shop, and entered, white and trembling, she found her little son dead, and the father lying senseless by his side.

### Chapter III.—Hospital Life.

THE RECEIVING ROOM.

The hospital, a large and busy one, whose doors were open at all hours to accident cases, and whose average was over eight hundred per month, was not very far away; and thither little Eben was borne, uttering as he went shrill cries of pain and distress.

"More fright than anything else, I expect," said the doctor shrugging his shoulders, "and perhaps a few bruises. Well, take him off to the Children's Surgical, and we'll see about him later on. Shouldn't wonder if he's deaf, by the way," and he clapped his hands, now a little way off, now close to the child's ear. "Ah, I thought so."

THE CHILDREN'S WARD.

So Eben was carried off into a large and very bright-looking room, in which were rows of little white beds, their little occupants so busily engaged with milk and bread-and-butter that they only found time for a glance or two at the new arrival. He hardly looked at them, and cried as loudly as ever when a young nurse, with bright dark eyes, took him in charge, undressed and washed him, and tucked him up in one of those little white beds. Children seldom cried when Nurse Jennie had the charge of them; her ways of talking to them were so bright and arch, and yet so gentle. But poor little Eben was for ever shut out from that comfort in pain and distress, the soothing influence of a human voice. Moreover, he might not have understood, could he have heard, all that she said to him, by reason of that cloud on his poor little mind. But she was not to be beaten in this way. Finding that he still cried, and refused the bread-and-butter which she brought him, she knelt down and began to kiss the little forehead, placing herself a little behind him, so that he could not see her. This reminded Eben of his mother. The sobs ceased, the big eyes closed, and in a few minutes, tired out with his long cry, he went off to sleep.

HAWKINS AS CARETAKER.

By the time his mother came, he had had a sleep, and some milk and bread-and-butter, as much as he wanted of either; and this was such an unwonted treat that his little face looked quite bright and cheerful. At first the mother seemed heartbroken, telling between her sobs that she had been forced to carry home a bundle of sacks, and that her husband, who was out of work, had promised to take care of the child during her absence, but (she supposed) had tired of being indoors, and slipped out to his usual haunt, shutting the door behind him. "And however the child got out, I can't say," she continued; "but he must have, somehow or another, and went right off after his father, he did—bless his little heart!"

ANGULAR CURVATURE.

The Sister in charge of the ward, into whose sympathetic ears were poured many such tales, comforted the mother by telling her that they did not think the little boy was much hurt, and that he would probably be sent home again to-morrow. But when the bruised back was examined, something more serious than bruises was discovered. The child was suffering, and must have been suffering for some long time, from angular curvature of the spine. No wonder he had never been able to walk far without resting that poor little mis-shapen back of his.

So instead of taking back her little boy on the following day, Mrs. Hawkins heard that he would be kept in the hospital for a month's rest and treatment, and heard it with mingled pain and pleasure. So she went home empty-handed; and little Eben settled down to his life in the children's ward.

JOYS AND TOYS.

After a time, that is. He mourned for his mother every night for the first week, and sometimes in the day, too; but everyone was so kind, and everything looked so cheerful and bright, that before long he had forgotten his troubles in his unwonted joys. There was a beautiful fire—such a fire as he had never seen before, burning away not very far from his bed, and keeping the room delightfully warm. The walls were painted in pleasant, restful colours; and over the line of beds opposite to him were pretty pictures. All the windows were adorned with richly-coloured autumn leaves, artistically arranged between the double panes of glass by the Sister herself; and by one of these windows stood a case of lovely ferns. But what Eben took most heed of were the toy animals which stood here and there: a cow who looked as if she were waiting to be milked, a rocking-horse, and a big white swan who went on wheels, and would accommodate any child who was so disposed between her wings. These and many other toys were shown off to Eben by a tiny boy with a bound-up head, who was allowed to run about the ward; and although Eben could not hear what the showman said, he watched his every movement with much interest.

NURSE AMY.

As for the nurses and "Sister," he took to them at once, and would follow them about with his wistful eyes, as though asking for the caresses which they, knowing he was shut off from tender or merry talk, were specially ready to bestow upon him. Nurse Jennie was his first friend; but he became almost equally fond of Nurse Amy, who was a great contrast in appearance to bright-eyed, merry-faced Jennie—pale, quiet, and worn-looking, with a mouth that told of suffering patiently borne. They were fast friends, these two young nurses, although so different in disposition and manner; it seemed as though the one, with her alert, untroubled mind, and keen interest in all that went on around her, brought life and hope to the other, who, young as she was, looked as if the spring of her life had been broken for ever. Until she entered the hospital, her whole energy had been devoted to the mere endurance of suffering, and she had well-nigh sunk under its weight; but this new work of caring for others had helped her to forget herself and her suffering; and now the loving devotion of her colleague came as an added and most unlooked-for help. The wounded heart, so cruelly deceived and outraged that it had even lost its faith in there being any true love anywhere, was slowly being healed.

SMILES AND TEARS.

A children's ward is a strange mixture of tragedy and comedy, of laughter and of tears. In one little bed a pretty dimpled child plays with its doll or its soldiers, or bubbles over with delighted laughter at some sally of a student's, or of its own. In the next will be a poor, wan, wasted little creature, looking as though it had never smiled in its life; or a little fellow moaning with the pain which all the nurse's tender care cannot entirely remove; or again, some grave, quiet, patient child, with a sad weight on its nervous, sensitive little mind—the knowledge that an operation is at hand.

Eben was a pathetic little figure, too, in his way, suffering but little, but evidently very low in vitality, and ever apart from his little neighbours by reason of his deafness. He never laughed, and but rarely smiled; but when the smile did come, it was of such beauty that it won all hearts. He could not be persuaded to play with the students, and would not even look at them until he observed that Nellie, the little girl in the next bed, made great friends with them; then he would watch their games, and occasionally break into one of his rare smiles; but never would he indulge in a game on his own account.

"BLOWING HERSELF OUT."

Little Nellie had been brought into the hospital badly burned, and was never tired of hearing the nurse recount to visitors how she had been lighting bits of paper at the fire, while Mary was at school, and mother and the baby were in bed ("and Jine, too—Jine was in bed," she would put in with severe emphasis if this important feature in the narrative were omitted), and how, finding her clothes on fire, she had done her best, poor little mite, to "blow herself out." She would listen, too, with great gravity, while it was related how on the first night, when her poor little injured arm had been bound up, Nurse Amy had suggested that she should say her prayers, and she had reproachfully asked, "How *can* I say my prayers when I've only got one hand to say them with?"

THE HOSPITAL BOGEY.

If Eben was interested in her, she was almost equally interested, though in a different way, in a companion in distress; a poor little thing of two years old, so badly burnt that it had been entirely swathed in cotton-wool. Not an atom of its face or head was visible, excepting two little burnt and discoloured lips, for which a slit had been made, and from which came now and then weak moans or cries, or a tiny pathetic little murmur, "Dop o' dink—dop o' dink!" Nurse Amy was constantly by its side, giving it milk out of a glass squirt, or talking to it in low, sweet tones, which it would stop its moans to listen to; and many eyes filled with tears at the sight of that poor baby, so young, and yet so suffering. But Nellie could not divest her mind of the idea that it was a "bogey"; and, though it was several beds away, and the nurses often tried to explain to her that it was to be pitied and loved, not feared, she would still creep to the end of her bed when she could, and peer anxiously out, to make sure that the poor bogey still lay there, and showed no symptoms of wishing to molest her.

TOMMY.

Just opposite Eben was the queerest little fellow, with the most roguish smile imaginable, and a quickness of wit which, considering his years—they were but three—was truly astonishing. He was suffering from double hip disease, looked wan and pale, and spoke in a weak little voice; but for all this he was brimming over with fun; the weakness of the little body only seemed to make the brightness of the mind yet more apparent. A lady friend of the Sister's was just then painting some exquisite sprays of flowers on the softly-tinted walls; and when she came to Tommy's bedside, and bent down to speak to him, these are scraps of the conversation that ensued.

"Well, little curly-head, what's the matter with you?"

"Why, nothin'."

"Nothing? Dear me, what did you come in for, then?"

To this he vouchsafes no answer; but when she presses for one, the bright eyes light up with a sudden thought,—"Ah! come to look at the ladies, didn't I?" And with all his mischief, he is anything but pert. The little mouth, whose smiles show a lovely set of pearly teeth, is gentle and sweet, as well as roguish, and he evidently expects the lady to rejoice with him at the little hits that he makes.

"I often wonder what the queer little thing would become if he lived to grow up," said the Sister with a smile, when Miss Maple, overcome with amusement, sought her out, and described this little passage at arms. "He would have made an excellent comic actor or singer some day, I should think. There is a winning little manner about him which makes all his fun simply irresistible."

"Then he won't live to grow up—are you sure?"

The Sister shook her head; and Miss Maple went back to his bed with a sigh, and kissed the little lips that were equally ready with a kiss or a quip.

---

### Chapter IV.—Nurses and Patients.

### A TALK WITH THE SISTER IN CHARGE.

Miss Maple was keenly interested in hospital life, and picked up much information while she was beautifying the ward with her brush, sometimes studying the children, sometimes talking with the Sister.

"It isn't quite so sad as I thought it would be," she said one day. "The first time I came in, do you know, the tears would rush into my eyes every minute: and it always must be dreadfully pathetic to see these poor little things, who should be romping about in the sunshine, lying here day after day. But perhaps it is generally more pathetic than dreadful. What do you think?"

The Sister smiled and considered a minute. "Of one thing I am quite sure—there is a great deal of happiness in this ward," she said. "We have very sad cases now and then—that goes without saying; but as a rule science and care can do much, very much, to alleviate pain, and to sweeten and cheer a fading little life; and I confess the thought which most often comes into my mind as I look round the ward is this: How much suffering is *spared* to these poor little children! What their lives might be—what their lives sometimes are—without proper attention, without proper food or nursing, is so terrible to think of, that I am often overwhelmed with thankfulness rather than sorrow when I look at them. What chance would they have at home, with mothers who may or may not be kind to them, but who have no notion how to treat them, and will hardly listen if you try to tell them? Look at little Eben here. We can't keep him more than a month, poor little fellow; indeed, I don't know that it is fair to others to have him so long; but it is evident that he has been practically half-starved at home, and we can at least do something to remedy that, and give him a chance. It is not his mother's fault, mind; she is evidently very fond of him. But they are poor and shiftless; and as for understanding how to look after him—just listen to this. You were remarking the other day what pretty fluffy hair he had; how it stood out round his head like an aureole. Well, when his mother came, the day after he had been brought in, she hardly knew him. 'Why, whatever have you been a doin' to his 'air?' she cried. 'It was always straight and brown, and lay quite close to his 'ead, it did!' And it turned out that the child's head had never been washed since the day of his birth."

"Dear, dear! the poor little boy will have but a poor chance when he goes home, I am afraid," said Miss Maple. But the Sister did not respond; she had gone off into a brown study.

### WORK FOR OUTSIDERS.

"Do you know what my counsel to the public would be, if they cared to ask for it?" the ward Sister broke out presently. "I would say to it but one thing. Supplement—supplement. We have our work here, and plenty of it too. We take child after child, and do what we can for it during the little time that we can spare. Then its place is taken; and so far as that child is concerned, we have done with it,—till perhaps it appears again, very likely worse than before. Ours is only a little bit of the work, after all. *We* put a child in a fair way to get well; someone else must go on with the work. *And*—someone else should prevent there being so much of it. If ladies like you, Miss Maple, would come into our hospitals for a year or so, and then go about among the people, using the knowledge which they have acquired, giving informal lessons on diet and the proper treatment of children,—ah! and pressing home the truth, which would have been impressed upon them, that the sufferings of these poor little children are often and often caused by the sins of their parents, I believe they might do a work as important to the full as ours. Look at the poor baby in the cot here; ten days old, and a mass of disease already. And that poor child in the women's ward, whom you asked me about yesterday; half-blind, half-deaf, and with her face half-eaten away by that loathsome disease that I needn't name. Look at them, and then hear the parents talk as if it was God's will that their children should suffer! Surely the time has come for *someone* to speak out!"

### HELPS FOR WEAKLY CHILDREN.

"But with regard to work that must come after, instead of aiming to lighten, ours," she went on, after a pause. "I don't mean to say that nothing is done. Everyone knows that convalescent homes are simply invaluable; and the Invalid Children's Aid Association is doing excellent work, too, though I don't suppose it is very widely extended as yet. Still the idea of giving each weakly child, as it leaves the hospital, into the charge of a lady who will visit it, and see what it needs, is a very good one. I like the idea, too, of an Invalids' Dinner-table, such as one of the hospitals has started. Through a ticket, or the payment of a very small sum, a child (or for the matter of that an adult) can get a good dinner there two or three times a-week; and that may be everything to him when he leaves the hospital, and goes into a home where the food will be always rough, and very often scanty."

### WHAT ARE THEY AMONG SO MANY?

"And you consider the work of incurable hospitals supplementary, too, I suppose," said Miss Maple.

"Certainly I do. Our hospitals are meant for the treatment of accidents and acute ailments. They cannot take chronic cases, and yet do justice to their own proper work. Here, again, I know very well that something is done. There are fifty beds in one incurable hospital, and I think about thirty in another. But what are they among so many? Practically, we have but two alternatives; to keep a chronic case, and so perhaps deprive several other children of the benefit they might have had, or to send it to a home in which it will but feed and develop its disease."

"But of course district nurses can do something for these poor children in their own homes; seeing to the proper dressing of their wounds, and so on?"

"Certainly, certainly. Their work, again, is very useful."

"MEAT EVERY DAY!"

But here the conversation was interrupted by the appearance of dinner; and for some time sister and nurses were hard at work, cutting slices off the joint, peeling potatoes cooked in their jackets, mincing the meat up small, and serving out cups of it to the little patients, who were eager and interested spectators of every stage of the proceedings. Then came the second course, delicious custard puddings; and several little crows or chuckles of delight were heard when they appeared. "Ain't it good!" Miss Maple heard one or two little voices exclaim; while one little girl, an old *habituée* of the place, leaned over to a new-comer, and said, patronisingly, "Yer like it, don't yer? And yer'll get pudden like this ere often, I tell yer, and meat *every day*. You think o' that!"

Meat every day! That is almost too great a stroke of luck for many of the little ones to be able to realise it, just at first.

CHILDISH JOYS AND SORROWS.

"You were asking just now whether I thought a children's ward very sad," said the Sister, as Miss Maple was gathering up her brushes after her morning's work. "In some ways I think it is less so than an adult ward. For one thing, children are so easily pleased. See what real pleasure this everyday event, their dinner, gives them. And then how easily their minds are diverted from their troubles!"

SHOT IN ITS CRADLE.

"Still, we do, of course, have cases which are most pitiful to witness. I shall never forget the sufferings of a poor little baby who was brought in one day, shot right through, from side to side. The father kept a loaded revolver on the top of a picture in the sitting-room—goodness knows what for; and the elder child climbed the back of the sofa and got it down, and, as ill-luck would have it, shot his little sister in the cradle. The poor little thing was writhing in agony, and the father was wellnigh distracted with grief. The mother, strangely enough, seemed to take it more calmly, and insisted on hunting up the child's grandmother and aunt, though I warned her the baby would not live many hours. We did what we could to relieve it; all that afternoon one or other of us had it in our arms; and before the mother came back it was dead. I hope I may never again see so tiny a thing in agony which might well tax the fortitude of the strongest man."

"NOW THE BOYS IS GONE, HE'LL GO, TOO."

"An accident! I expect two children will be brought in directly," the Sister said: and in a minute or two all preparations had been made for the new comers.

But their stay in the hospital was to be but brief. Inside those bundles were two little unconscious figures, so burnt and charred that they might have been taken for black rather than white children. Their mother had left them (so she explained) for but a quarter of an hour, while she went to get something for their dinner. When she came back the room was dense with smoke, and the two little boys, her only children, lay senseless on the floor. There was no hope for them; she knew it almost before the Sister broke it to her; and before six o'clock that evening those little charred figures had been borne away once more, now stiff and cold in death. Then the poor mother, who had been brave hitherto, broke down altogether, and dropping down on the stairs, clutched the baluster, and looked up at the Sister with utter despair on her face. "I've lost my all," she said; "my husband and my boys, all in one day. Yes, Sister, I know what I'm saying; it's true, it is. He'd a left me plenty times if it hadn't been for the boys. I've done my best to keep him, but he's always been against me these four years or more; and now the boys is gone, he'll go, too. I know it as well as if I'd seen it."

TOO LONG ON THE STRAIN.

During Eben's month in the ward the Sister's face became more than usually careworn, for she had many anxious cases, not only in the ward, but outside, too, there having been so great a pressure upon it that, even after passing on as many as possible into spare beds in other wards, the surgeons had been obliged to discharge several children whom they had much wished to keep longer. Moreover, she was troubled about Nurse Amy, who had been manifestly drooping of late, and who would, she much feared, break down altogether. The girl was evidently struggling on bravely; but there comes a time when the best will in the world can no longer breathe energy into the overtaxed body; and one day Jennie looked round the ward in vain for her friend—Amy had given way at last.

The two saw almost nothing of each other that day, and Jennie hoped that the rest was doing her friend good; but the next morning, she had entered the ward but a few minutes, when Amy came in, pale and exhausted-looking, but evidently resolved to go on as usual. She went from one bed to another, moving as though there were a heavy weight on her, and hardly speaking a word throughout the day.

A HAUNTING FEAR.

Later on in the evening, when the children had all been tucked up for the night, and the two friends were off duty, Jennie found out that Amy was tortured by the fear that her health would give way entirely, and that she would have to give up work.

"You don't know, Jennie, how sick with terror the idea of it makes me," she said. "While I am working hard I don't have time to think, and then I get so tired that I sleep all night, so that thoughts can't come then either. But if I had to just lie still, thinking of all the terrible past, and of what might have been, if only—if only——Oh, it makes me shudder only to speak of it! Even that day and night by myself, when I was thinking that this might be my life henceforth, were a torture to me."

"But you are not going to break down, my poor Aimée; I won't let you," said Jennie, stoutly. "You only want a little rest and change. You came here perfectly overstrained with all your troubles, you poor thing; and now the work coming on top of that has been too much for you. I must see what can be done."

So she consulted the Sister on the first opportunity, then sought the matron, and laid the case before her.

A REAL HOLIDAY.

"Poor child!" said the kind-hearted matron. "Work is good for her, no doubt, but she must have rest too. A nurse's work is certainly very trying, and, as you know, I often wish you could all have a little more change. That is why I welcome so gladly the tickets for concerts or theatres that are sometimes sent us, and why I wish so much that the public, who must often have boxes or seats going begging, would think of our hospital nurses more than they do. But these things are for such as you, Jennie, who are well and strong, and only want a little brightening up now and then. As for your poor friend, how do you think she would like this? She can't go home, you say—the journey is altogether too expensive for her. But a lady in a beautiful part of the country, and not far off either, has offered a home in her own house to one or two of our nurses at a time; and I think a week or two there would be the very thing for Amy—far better than going to some regular holiday home for nurses. To meet a number more nurses, and be tempted to 'talk shop' all day long, is just what one does *not* want in one's all too rare holidays."

"I should love to have Amy go," exclaimed Jennie. "Only I doubt if she has spirits and energy enough to go into a strange house alone."

"When is your time in the children's ward up?" was the apparently irrelevant answer to this.

"Next week, Matron; and then I am to go on night duty, you know."

THE HEALER HEALED.

"Ah, I thought so," and the matron smiled, and said no more just then. But when Amy went for her holiday, her bright-eyed little friend went too; and what with the rest, and the fresh country air, and Jennie's bright yet tender companionship, Amy became once more strong to suffer and to do, and took up her work—the best of all work for those who would forget themselves and their own afflictions—with the energy, not of despair, but of hope.

She had not been forgotten in her absence, although funny little Nellie had gone away, cured; and those big, wistful eyes of her little admirer, Eben, which had shed many tears at her departure, no longer looked gravely out from the little white bed. That bed had been wanted for some other child, and the Sister was obliged, reluctantly enough, to bid him farewell. They put him into a plaster-of-Paris jacket, by way of giving rest to the poor little spine, and recommended him to the care of one of the members of the Invalid Children's Aid Association; and then the dirty, squalid little room which had seen his birth claimed him once more for its own.

**Chapter V.—At the West End Home!**

HAWKINS' LITTLE GAME.

But it is time we took another look at the West-end home — the home which had been rendered childless, joyless, by that everyday affair, a street accident.

For some days the parents of little Lion were perfectly crushed by the suddenness of the blow. It seemed to them that there was absolutely nothing to live for; and they were sitting together one evening, idle, and in perfect silence, as was now their mournful custom, when the servant announced that a man named Hawkins wished to see them.

"Show him in here," said Julian listlessly, not even caring to move, except when he was obliged; and a minute later, Eben's father came into the room, slightly unsteady on his legs, and with a foolish smile, which was meant to be ingratiatory, on his lips.

"I've 'eared, yer see, sir," he began, "as 'ow you're the gent as had a child in that there haccident my little chap got into; and I thought as you'd like to 'ear they've took him into the 'orspital; and he ain't very bad, the doctors say, but they'll keep him there a bit."

Mrs. Heywood leaned forward at this. "O Julian, I've never thought again about the other child, since we heard he wasn't much hurt," she said. "But I am very glad to know it was true. And he is quite deaf, poor little fellow, isn't he?"

"Yes, mum, and idioty too. He never did seem——"

But here the man stopped, startled by a sudden move from Julian, who leapt to his feet, and setting his teeth, asked, "And you've got a lot more of 'em, I suppose, eh?"

ONE TAKEN—THE OTHER LEFT.

"Eight on 'em altogether," said the man with a grin; and, seeing that Mrs. Heywood looked more sympathetic than her husband, he turned to her, and maundered on about the trouble and expense of such a family, while Julian flung round, in an agony of misery and rebellion against the fate which had spared this child and snatched away his own. "Good God!" he ejaculated, and then stood motionless, with his arms on the mantelpiece, hearing, and yet not hearing, in the tumult within him, all that Hawkins was saying.

"GET OUT!"

"I am glad to think that this is no extra expense to you," Mrs. Heywood broke in at last. "Your child will be off your hands for some weeks, and better cared for than if he were at home."

"That's true, mum," admitted the man, reluctantly. "But there's all the worry—that tells on the missus, it does; and then there's goin' to see him at the 'orspital, and me losin' a day's work now and again to see after the missus, she's so upset like; and so, if you'd be kindly pleased —"

Julian turned round at that, in a white heat of passion. "I'll be kindly pleased to kick you out, if you stop another moment!" he burst out. "I've heard this much about you—that you sneaked off to the public-house when you'd undertaken to look after the child, and that it's through you the whole thing happened. You nearly kill your child, and then you think to get money out of us—to drink his health with, no doubt—I know your sort! Get out!"

The man vanished, and Julian, still trembling with passion, turned to his wife. "Get my cheque-book," he said. "Now address an envelope to the secretary of the hospital—you know its name—while I write the cheque. There, I send them five pounds for looking after that child; and now—never let me hear of him again—do you understand ?"

He spoke with such unwonted roughness, that she felt he was dreading lest she should desire, as indeed she did, to know more of little Eben; and bowing her head, she turned away, afraid even to shed tears, now that this strange mood had come upon him.

ON THE RACK.

From that day Julian Heywood seemed to become a different man. He was as though tortured by some invisible spirit, driven hither and thither, from one task to another, never allowed to rest for a moment, and yet, while working his hardest, in an agony of uneasiness and irritability. He was of a literary turn of mind, and had always had several irons in the fire which he would take up out of business hours; now he set to work with all at once, and toiled late and early, never giving himself any rest or relaxation, and working his secretary, a good, faithful, but somewhat slow man of middle-age, almost as hard as himself. Thomas Jenkins loved him as a son, and being of a meek, yielding nature, would stand anything from him; and there was something very pathetic in the sorrowful patience with which he would bear with Julian's unreasoning fury against him, bowing his head before the storm, and striving, as far as in him lay, to carry out the wishes of his employer. Mrs. Heywood dared not interfere; she seemed to have lost all influence over her husband, and as it was, often came in for a share of his ungovernable anger, careful though she was not to rouse it. If her heart had been sorely wounded by the death of her little son, she thought it must be breaking altogether now, under the weight of this new and terrible trouble.

A CHANGE FOR THE WORSE.

At last a change came. Nothing that the doctor said in the way of advice or warning seemed to have the least effect upon Julian Heywood; but one day—no one knew why—he announced that he should not get up to breakfast and that his secretary might go home. That proved to be the signal that he had dropped work, and after months of severe and incessant labour, he suddenly started off on an opposite tack, and did simply nothing. His wife hoped that it was merely the exhaustion consequent upon severe labour, but the doctor shook his head. "We must rouse him from this—give him change, interests of some kind or another," he said. "Get him to go abroad now; that might do him all the good in the world."

But Julian was as stubborn in his do-nothing state as he had been in his feverish determination to work. Nothing could rouse him from the dull torpor which had crept over him; and even the mention of little Lion, though it apparently sent him off into long musings, did not appear to stir any active emotion of any kind within him. His poor wife, though she did not lose the power to suffer, seemed to lose with him the power to hope. What had she left to live for? Her child had been taken away; her husband, once so bright and loving, cared for life—and she sometimes thought, for herself—no longer; what was there to look forward to but death?

"ONE TOUCH OF NATURE MAKES THE WHOLE WORLD KIN."

She wished indeed to keep up her health so far as she could, lest she might prove unable to wait on her husband; and with this object she took an hour's walk daily, though hardly noticing, and certainly not caring, where she went. In one of these walks she found herself close to one of the underground stations, and feeling very tired, sat down in the waiting-room to rest. A lady was there, with her little boy; and as Florence Heywood watched her arranging his cap and his curls, and bending to kiss the rosy face, a sudden hunger at her heart made the tears spring to her eyes. Mother and child soon passed out; and as Florence's wistful gaze turned from their retreating figures, she suddenly became aware of another person in the room, a poor but respectably dressed woman, whose eyes met her own, and like her own were bright with tears. In a moment Florence took in the fellowship and sympathy expressed in that glance. They had both been mothers; they had both lost a child; and their hearts warmed towards each other through the remembrance of the life-long burden that each alike must bear. No one else was in the room. With a sudden impulse Florence stole across to where the woman was sitting, put her hands on her shoulders, and kissed her; and then the two sat and wept together.

A GRATEFUL MOTHER.

Very soon Florence was in possession of the other's story, which proved to be one, not of grief merely, but of consolation. The poor woman could not speak gratefully enough of the care and kindness which had been lavished upon her little girl in the hospital where she breathed her last, and of the wonderful improvement, both bodily and spiritual, which manifested itself very soon after she had been sent there. "She was such a perverse, contrairy child at home," the mother said; "it would almost break our hearts sometimes to find how nothing we could do would please her. But after she'd been there a week or two, it was like heaven to sit by her bedside, bless her. Of course, they nursed her ever so much better than I could; and then they was so kind and wise-like somehow, and talked so nice to her, she couldn't but listen. That nurse of hers was an angel, if ever there was one, and I tell her, 'If my Bessie's an angel now—and I do believe she is—it's all owing to you.' 'Under God,' she'll say to me, as sharp as sharp, whenever I tell her that. Because I go in pretty often, and I take some flowers to the poor little dears, and sometimes I bake 'em some little cakes, and carry them with me, for we're not so bad off now, and it does me good like to do what I can for 'em, and to think it's along of her I'm doing it."

"How nice! How good of you!" murmured Florence, pressing her hand.

THE DAWN OF HOPE.

"Why, you see, ma'am, it ain't good—it's just a pleasure like," the woman answered. "And 't ain't only me as does it. There's others as 'll bring little things in to the children, so the nurses tell me—people who've had a child get well there mostly, and know what a lot of good them hospitals do. Sometimes I can't but cry a bit to myself, and think if only *I* was like them, giving things out of gladness, as you may say. But there, the sorrow's come; and going there don't make it worse—it just lightens it."

A faint ray of hope seemed to dawn upon Florence's heart as she listened to the woman's simple tale; but now a sudden pang shot through her as she thought how she, too, might have given out of gladness—how she would have given anything, everything she possessed, if only her little darling had been spared to her. But the pang passed away, and the little ray of hope remained. She bade an affectionate farewell to her new friend, and wended her way home, full of thought.

FLORENCE'S PLAN.

The next day she was out considerably longer than usual, and came back looking tired and anxious, but with a certain subdued excitement in her eyes. Her husband did not notice it—he noticed hardly anything now; and all that evening her anxious eyes kept turning to him, as if she were trying to realise what effect a projected course of action might have upon him. At last she rose, and, coming close to his chair, knelt down beside him.

"It's my birthday to-morrow, Julian," she said, wistfully. "We always used to make a fête of my birthday, didn't we?"

Something in the words or the tone seemed to rouse him a little, and with now unwonted tenderness he stroked the once flower-like face, now thin and lined with sorrow. "'Once—in the days beyond recalling; Once—in the golden days,' as you used to sing," he said sadly. "Life has no more fêtes for you, my poor little Florence, nor for me—nor for me."

"And yet," she said, again with that wistful look, "I should like to go out somewhere to-morrow. Only you must go too—I could not go without you to take care of me. Will you, darling? You would like to do anything you could to help and please me," for he was now considering her sorrow-lined face, with tears in his own eyes—the first she had seen for months. "And I know this will do me good."

SO FAR, SO GOOD.

"And what am I to do then?" he asked, looking puzzled, but not uncompliant.

"I will show you to-morrow. You shan't be bothered in the least about the journey, darling, and I won't say a word more than thankyou, to-night, because I know you're so tired. Let me read to you, shall I?"

He dropped the subject at once, falling back into his old apathetic calm, except for a certain tender observance of her face, which seemed to her a good omen. She read him to sleep; and then, still holding the hand he had stretched out for hers, bent low over it, and in an agony of mingled hope and fear prayed for the success of her plan.

### Chapter VI.—Existence at the East End.

EBEN'S HOME.

Little Eben got on pretty well for the first few weeks after his return from the hospital, though he sadly missed the good food which he had been used to there. The lady into whose care he had been confided visited him regularly; and this of itself was a great thing, making his mother more careful than she might otherwise have been about his cleanliness, and providing him with various little enjoyments which only the "leisured classes" have time and opportunity to devise for the sick and helpless. But Hawkins had less work than ever now, owing partly to his unsteadiness, partly to general slackness of trade. Various difficulties pressed hard upon the family, and at last, seeing no other way of escape, they suddenly "flitted," taking what was, for people of their class, quite a long journey, and settling down in a still more squalid neighbourhood and a still smaller and dingier room. The "visiting lady" lost all traces of them; and now, with work as slack as ever, and no outside help of any sort coming in, the family sank lower than ever before, little Eben, as the most weakly among the children, feeling the change most of all.

There were now five little ones, besides the prospect of another; and one evening, when the mother looked round upon her children and thought of her troubles, present and to come, she broke down and sobbed, even little Eben's caresses failing for once to comfort her.

"ALL ALONG OF SEEING THE HOME."

That afternoon there had been an unusual stir in the house. A woman living in a downstairs room, the wife of a man who earned good wages, but kept her and the children terribly short, had gone, two or three weeks before, to a Mothers' Home, and had now come home again—not as she went, alone, and so weak with hunger that the basin of milk gruel which she ravenously caught at proved to be more than her weakened stomach could stand; but by her husband's side, in a cart which he had borrowed for the purpose, her baby in her arms, and her cheeks no longer hollow with want and care. All the neighbours came to have a look at her, and to Mrs. Hawkins she confided that her husband's visits to her in that cosy little room at the Home (for the Lady Superintendent had pressed him to look in every night), with its bright fire and clean white bed, seemed to have made a different man of him. "And when he come for me this afternoon," she went on proudly, "he says to me, he says, 'You won't know the room again. I've washed the bed-clothes and made the bed, and I've scrubbed the room, and lighted a fire with some coals I got on tick; and there's some gruel on the fire ready for you to take as soon as you get home. And its all along of seeing the Home; so now come on, old gal, and thank the lady for all she's done for us.'"

Many more particulars followed, of the good nursing and food at the Home, and the "'eavenly quiet" of it; and when poor Mrs. Hawkins looked round her own dirty, crowded, noisy little room, and thought of all she had to go through in it, it was hardly any wonder that the tears came.

"I think it'll drive the clean out o' my mind this time, or else kill me; and I wish it might do that," she sobbed out at last; "for there's no comfort for me here. Only how the poor little 'uns 'd do I don't know."

HAWKINS RESOLVES TO "GO AND DO LIKEWISE."

Hawkins was sitting at the other side of the chimney-piece, smoking, and he looked up at this. He was by no means a bad-hearted man, when not in liquor, and just now he had been sober for several days, for the simple reason that he had had no money to buy drink with. "Don't cry, old gal," he said; "I've been a bad husband to you, and you've had a lot to put up with. But if you can get into that Home, you shall—there! And I'll get along somehow or another, so's you shall have a good time for once, see?"

It was a delightful prospect, except for the difficulty of leaving the children; and Hawkins could not rest till he had gone straight away to the Home, and asked what steps he must take to get his wife admitted. The cheery Lady Superintendent, who was on the steps, informed him regretfully that there were but eleven beds, and that those were booked for months to come. "So much work, yet such a little bit of it can be done—and even about that we have great anxiety as to ways and means," she said, sadly. Then, seeing the disappointment on Hawkins' face, she added: "But I'll tell you what I can do. I see you're really anxious about your wife; you haven't thought about her much hitherto, and now you're going to do better, is that it? Ah! I'm glad to hear it. Well, you send for me, and I'll come and attend her in her own home. And mind this: If you do all you can to make her comfortable, and to keep her mind easy about you yourself, it won't be such a bad time for her, after all."

"I will, I will, ma'am. I'll do my best for her," said poor Hawkins. And he meant it.

LIFE AND DEATH.

But, alas! he had little opportunity of carrying out his good resolutions. Two or three weeks before the baby's arrival, he caught a chill, and became seriously ill; and when the Lady Superintendent answered her summons, it was to watch one life leave that crowded little room just as another was about to enter it. In one short hour, poor Mrs. Hawkins, worn almost to a shadow with grief, with nursing, and, alas! with famine, became a widow and a mother.

---

### Chapter VII.—Tired Out!

IN THE CHILDREN'S HOSPITAL.

We have not time to dwell on the terrible condition to which the Hawkins family had been reduced. Suffice it to say that little Eben was found to be not only wan and wasted, but suffering from abscesses on his poor little mis-shapen back. Partly from ignorance, partly from the multiplicity of her other cares, his mother had neglected to attend to him properly; and now, when the plaster jacket was taken off, he was seen to be in a sad state. The hospital was evidently the only place for him; and being taken as an out-patient to one specially for children, and not very far distant, he was speedily admitted.

What a change for the poor little fellow! Here, though he must still suffer, everything was done for him that could be done. Kind faces bent over him; kind hands brought him delicious food, and as much of it as he could take; everything was sweet and bright and clean; and merry little faces of children who were well enough to get up and dress, peeped at him now and then, and won from him one of those smiles which had of late been never seen on the poor, pain-worn little face.

EXIT—THE NURSES.

One afternoon, about a week after Eben had come into the hospital, there was a rather unusual stir in the ward in which he lay, as though in expectation of some event. Not that any special preparations were made—everything was always spick and span any afternoon that you might choose to look in; but the nurses were bustling round (if such a word may be used to describe their quick, light movements), peeping in at every cot, in case anything might be wanted by its little inmate, putting out toys, and making such preparations generally as might prevent their being wanted for a quarter of an hour or so. Then, at a signal from downstairs, they slipped across the passage and into the bright little ward-kitchen, leaving the ward to take care of itself. A minute later, steps were heard on the staircase, and a lady and gentleman came to the threshold of the ward, she eager and trembling, he drooping and languid, and apparently taking little heed of anything around him.

ENTER—FLORENCE AND JULIAN.

It was not the first time that Florence Heywood had stood looking down that long vista of little white beds, with childish faces peering brightly out, or lying back, white and wan, on the pillows. She had come, the day before, to confide her plan to the authorities there, and to ask for a little help in carrying it out; and when she had stood, as she was doing now, at the door of the ward, she had burst into tears, not merely at the world of tender memories which it brought before her, but for the very pathos of the scene itself. Now, however, she was too overwhelmed with anxiety as to the effect which it might have on her husband, for the blessed relief of tears. Pausing but for a moment at the door, she led him hurriedly in, saw the sudden flush and spasm which passed over his face—as though he had received some sudden and painful shock; and then, her strength giving way altogether before the terrible fear that she might have done him harm rather than good, she sank down on the end of the nearest cot, white and trembling, and with a sudden darkness before her eyes.

"Is oo ill, lady?" a tiny voice beside her asked; but she heard nothing, saw nothing, until a hand was laid on her shoulder, and a husky voice said, with some reproach in it, but no anger;—

"Florence, why torture yourself—and me—in this way?"

"WE WANT TO REMEMBER HIM—IN A HAPPIER WAY."

She looked up at that, saw that his face was working with emotion, and rising, drew him to the window, where they stood together, she clinging to his arm. "Julian, forgive me, forgive me!" she murmured, her tears falling fast. "I know it hurts you—makes you think of—of—" she could get no further with that. "But we want to think of him; we want to remember him—in a happier way; out of our loss we might make others' gain. O Julian, we have lost our little child; but we may yet have children to care for; let us open our arms to them, and see our little Lion once more in them! Shall we, Julian—shall we?"

"What could we do?" he asked, almost harshly; but Florence looked at his face, and knew that that was the only voice he could command just then. She pointed for answer to where, above a little bed just opposite, was a gilt scroll, "In Memory of dear little Arty," and above it (she had not noticed that before), "Inasmuch as ye have done it unto one of the least of these my brethren, ye have done it unto me."

"Unto Christ, and to our little angel Lion," she said softly. "Don't you remember, Julian, how he would distress himself over the rags and poverty of children in the streets? And he died—oh! my little darling boy—as he had lived, trying to help others. O Julian, we have been terribly selfish in our sorrow, as in our joy. Let us begin a better way. Let us make those last words of his come true—'Lion 'll help you.'"

"MUCH BETTER, THANK YOU."

It was with the greatest effort that she said as much as this; now she broke down, and they wept together, but with tears which were less bitter than any that had been shed since Lion's death. The thought of making her own grief into an instrument for working the good of others seemed to have taken away much of its bitterness, as far as Florence was concerned; and she was not without hope that the same consolation was working in the heart of her husband. She would say no more just then, however. Wiping away her tears as soon as she could, she gently soothed him, then asked if he would not just have a look at the children, and took him first of all to the little girl on whose bed she had sat, and who held up her face gravely for a kiss. 'Is oo better now, lady?" she asked, and Florence answered gratefully, "Much better, thank you, darling."

FROM BED TO BED.

They went from one bed to another, now watching a poor little wasted thing, as it lay sleeping with its head on its hand; now saluting a rickety baby, with an enormous head, who gravely extended a hand to each; now feeling in their pockets for some harmless little article which would gratify a curly-haired child, who suggested, when she saw Florence's watch-chain, "'Haps dat chain's for me." Now they sighed over a tiny skeleton of a baby, more like a monkey than a human being; the next minute they could not but laugh over a funny little fellow, with his face half hidden in plaster (he had fallen over a stone, he told them), who tugged at Florence's hand, and having thus attracted her attention, scampered off to pull string in a great toy bear, and force from it a blood-curdling growl.

AN OLD HEAD ON YOUNG SHOULDERS.

The set of beds in which the boys were placed was divided off by a screen, and here there were several sad cases, as well as several cheering ones. Some of the boys were calling to each other from bed to bed, having evidently started some game; but one who lay between two of these merry youngsters was lying back and crying quietly to himself. Florence sat down by his side, and soothed and petted him; and when she moved away, he looked after her with such a wistful gaze, that she turned and said, "I will come again, dear boy. I may, mayn't I, Julian?" He nodded, and then drew her off to listen to the tale of the biggest boy in the room, who gave his name as "Dan'el," and told very solemnly how he had been run over, and was brought in with concussion of the brain and a twisted arm. He looked grave and anxious far beyond his years; and Julian did not wonder, when he heard how the father was also in hospital "with pleurisy, and digestion of one lung," how the mother, too, would often spit blood, and how the boy feared he should lose the work which had been almost all he and his four brothers and sisters had had to depend upon since the father was taken ill. "And the doctor says I'll be longer gettin' well 'cause I hadn't had much to eat afore I come in," he finished up mournfully.

A CHANCE MEETING.

As they passed a bed further on a little face caught Julian's attention—a little face with big wistful eyes, and crowned with a glory of fair hair; and as he looked, a sharp thrill ran through him. Catching at his wife's arm, he went with faltering steps towards the bed, and there, on the card at the foot of it, was the name—Ebenezer Hawkins.

Florence gave a cry of something like dismay. "O Julian, I never knew it; indeed, I never knew it!" she said, trembling again for dread as to what the result of this new shock might be.

But her husband only gazed a minute at the child, and then turned away. "We will come again, Florence," he said, in an unsteady voice. "I am so tired; I—I don't think I can bear much more."

She hastened to follow him, and when they were once more in the cab, he fell asleep, evidently tired out.

### Chapter VIII.—Something to Ponder Over.

FLORENCE'S BRIEF.

Nothing more was said on the subject that afternoon; but late in the evening, Julian, who had been silent as usual, but with a silence which Florence could not help hoping was of a different kind, suddenly roused himself, and stretched out his arms to her.

"My wife knows how to make cunning plans of her own," he said, but with none but mock reproach in his tone. "We are to devote ourselves to children's hospitals, are we? And what further preparations has she made? Is the brief all ready? Come, let me hear it."

He spoke lightly, almost playfully, perhaps trying, man-like, to avoid a further display of those deeper feelings which had shown themselves that morning. But Florence could not answer him in the same strain, "Only—only that I want you to do it, darling, for Lion's sake," she sobbed, hiding her face on his shoulder.

A very woman-like brief; but it was all that was needed just then. "And I will—I will," he said tenderly. "There, don't cry any more; it is bad for you, dear. And we don't want to cry; we want to think and to do. I will prepare the brief, shall I? I will just make a short study of the whole question—go round and look at the hospitals, and find out what they do and what they need—with you to help me, if you like. And then we will see what we ourselves can do. At any rate, you shall have a cot in the hospital we went to-day; that I can safely promise."

STUDYING THE QUESTION.

And he was as good as his word. The idea of taking up the subject, and going thoroughly into it, seemed to put new life into him, after those weeks of miserable apathy and depression. As health and spirits returned, he began to take up once more his regular work; but the interest of his new study never flagged; and one day, not many weeks after their first visit to the hospital, he called his wife into his study, and producing a sheaf of notes, told her, half laughingly, but with deep earnestness underlying his light manner, that the brief was ready—would she come and listen to it?

"First of all," he said, throwing a paper into her lap, "there is a list, as complete as I can make it, of the children's hospitals in London. Of course there must be thousands of children treated every year in other hospitals too; and as long as they are in a children's ward they do very well. To mix them up with adults seems to be a very unfortunate arrangement, partly on account of the children, who, if they are small, have to be constantly hushed down, and if they are bigger, hear and see many things not suitable for them; and partly for the sake of the other patients, who are worried alike by the cries and the laughter of the children, and generally come into a hospital needing rest and quiet almost as much as anything. And yet I don't see what is to be done, when, as often happens, the children's ward is full to overflowing, and urgent cases still clamour for admission. Really, the only remedy for this, and for general overcrowding, which presses very hardly upon nurses and doctors, is for hospital accommodation to keep pace with the times; and that, of course, it can't do without the generous support of the public. But I beg your pardon, Florence; it was of the list of children's hospitals that I meant to speak just now.

DROPPED FOR LACK OF FUNDS.

"Here they are, you see, with their names and addresses; I have just jotted them down in the order in which we visited them. Number One, you see, is the hospital you lured me into first of all. It serves for the north-eastern part of London, has been at work for twenty-three years, and seems, from all I hear, to be excellently managed; but for some reason—possibly because of its not very attractive name—it appears not to be very widely known. Did you hear the Lady Superintendent say the other day that the convalescent home attached to it had had to be dropped for lack of funds? And although the need of more accommodation is great, and a piece of land has been given for the purpose, they have at present *no* money in hand for a building fund. Here is a case for help, don't you think so?

WANTED TIME AND TASTE—AS WELL AS MONEY.

"Number Two on the list does duty for East London, you see, and an excellent hospital it is. Here, instead of medical and surgical wards, they have a Boys', a Girls', and an Infants' Ward—you remember the number of poor little half-starved mites in the cots in that ward, don't you?—and one side of each ward is 'medical,' the other 'surgical.' True, medical cases generally need a higher temperature than surgical ones; but I suppose hospital authorities have but a choice of difficulties, for it would be impossible to make all the cross-divisions which might be suggested. In a perfectly ideal state of things, I suppose small children should be divided off from big; and there certainly seems to be some advantage in dividing girls from boys, though in other hospitals the difficulty is, for the most part, got over by screens, such as we have already seen, and, of course, every bed has a little screen of its own, which can be used when wanted. The out-patients' department of this hospital is an important feature in its work; and they tell me there is urgent need for more accommodation there. As for the in-patients' part, we both thought the wards charming, didn't we?—with their flower pictures and artistic wall decorations. After all, it isn't only money, as some people think, that is needed in hospitals: time and taste, if only they would give them, would do much to make the lives of the poor little patients, and the nurses (why should they be forgotten?), brighter and happier.

A WHOOPING-COUGH WARD.

"Numbers Three and Four are in the south-eastern district, you see. I don't think you went into the whooping-cough in Number Three; you would have liked to see a tiny, waxen-faced baby there, looking for all the world like a good-sized doll, and to hear that one or two of the children, who were all but suffocated when they came in, were fast getting well. Number Four doesn't take accidents; but on the other hand, it does what I haven't heard of any other hospital doing—that is, its medical officer attends cases, within a certain area, in their own homes.

"Then there is a north-western hospital, which, by the way, has adult as well as children's wards, but whose special object is the treatment of sick children. Its eighteen cots can't be sufficient to supply the needs of the district; but even as it is, I'm afraid the committee have much cause for anxiety as to ways and means.

A DEPARTMENT FOR PAYING PATIENTS.

"Next comes a hospital for the south-west—you remember its pretty garden, and the conservatory at the end of one of the wards. A special feature there is a separate building in which the children of parents who can pay are taken. It was only started last year, so that one can hardly tell yet how it will answer. I know you don't think it will be used, because you say ladies always want to nurse their own children; but no doubt there are cases in which they can't do it; and again, for children coming up from the country, such an institution might be found very useful. So that I think it is worth a trial.

ON THE ALERT.

"Last, but not least, comes what I think must be called the pet children's hospital in London. It claims to be older than any other, and has a good many patients from the country—some even from abroad; and although there are already 127 beds, it greatly needs more, and hopes to add a new wing, after which it will be able to take in accidents, which it cannot at present do. It seems to be under very energetic and competent management; and indeed, so far as I can see, all the hospitals are on the alert, providing isolation wards, replacing floors which require constant scrubbing by polished boards, turning their accommodation to the best account, and trying in all ways to make themselves as efficient as possible—or rather, alas! as scanty funds will allow. There, have I tired you out, Florence?"

A CHILDREN'S HIP HOSPITAL.

"Not a bit. In fact, I was just going to remind you of a hospital which you seem to have left out—the one where the poor little things were all on their backs, with hip disease, you know. I don't know that any hospital was sadder than that, at the first glance, at any rate; still, many of them seemed wonderfully bright, in wits as well as in spirits. Did I tell you that the boys were quite little politicians, and as eager as any schoolboys about the Oxford and Cambridge boat-race? Some of them were playing with soldiers, too, and seemed to take such an eager interest in everything connected with a soldier's life. Poor little fellows! such a life must be closed for ever to them, I

am afraid, even if they get well, as I believe the hospital claims that many of them do."

THE REST CURE.

"To be sure it does. They consider there that perfect rest for two years or longer is of more use than any operation; and certainly, when one hears that the children are kept sight of for years after they leave, it would seem that the permanent cures reported must be real. I only left this hospital out of my list because the children stay in it so long that it seems in some respects more like two hospitals, or rather homes, that we must see later on. In fact, I feel great sympathy with it, and all the more so, as I am afraid from what I hear that it is sadly in need of help just now."

"SO MUCH TO DO!"

Florence clasped her hands together in perplexity. "So much to do—so much to do; and we ourselves can do so little, in comparison with all that is needed!" she exclaimed. "What shall we do? Whom shall we help? But I suppose, after all, we had better wait to decide until we have seen the other two Homes you speak of."

"I think so, too, dear. For one thing, we have to consider what can be done for that poor little Hawkins. They tell me at the hospital that they cannot help him—he is quite incurable; and as they truly say, a hospital is not the place for incurable cases. His mother, as we know, cannot take him; she has a hard enough struggle to maintain herself and the other children; and, besides, she is quite unfit to have the charge of a painful case like this. We had better visit the Incurable Homes, and see what chance there is for him there, don't you think so?"

### Chapter IX.—The End of the Matter!

SICK AND INCURABLE.

The next day Florence and Julian visited a Home for Sick and Incurable Children, a handsome new building containing several large and cheerful wards, two of them with fine views across and down the river. Here were some fifty little patients, suffering from paralysis, hip and spinal disease, malformation, and various other ailments, and when Florence heard that some cases brought in as incurable were successfully treated, she longed to see little Eben there. It was not the place for him, however; that poor little clouded brain of his rendered him ineligible; and so the Heywoods bade farewell to the Home, though not before they had heard of the need for increased funds which the enlargement of the Home had necessarily brought with it.

"Another item to be added to our list of Children's Hospital wants!" sighed Florence. "It will be full to overflowing soon; and so, I think, will my heart."

ANOTHER INCURABLE HOME.

They went on at once to the only other Incurable Home they knew of, and here at last seemed to be the fitting resting-place for Eben, during the few years which still remained of his suffering little life. There was no difficulty about the payment—Julian was ready enough with that; but just now there was no vacancy, and Eben would have to wait till a poor lad should attain the age—sixteen—at which, by the rules of the Home, he must leave. The Lady Superintendent's eyes filled with tears as she spoke of this approaching event.

A SAD PROSPECT.

"You don't know how my heart aches when I think of it," she said to Florence. "I have been praying and praying—I hope it isn't wrong—that he might die before he had to go out (many of them do about that age); for we have nowhere to send him to but his own home, and I know too well what that home is, and how terribly he will feel the change, after all the care and kindness he has had here. It seems as if it was preying on his mind, and I don't wonder. A year ago we sent another boy home; we had got two or three already into various Homes (with a big H, you know), and it was no use trying for him too; and when we sent to see after him, he was so altered you would hardly have known him for the same boy. I simply could not bear it, and we managed to beg money enough from friends to send him to a Home for a year. He is ever so much better now; but the year is nearly up, and what we are to do then I don't know—I am ashamed to beg any more."

THE MISSING LINK.

"But can't they be got into Adult Incurable Homes?" asked Julian.

"Not till they are twenty-one or twenty-two; and then, of course, there is much difficulty about it, as I daresay you know. However, what we so dreadfully need is some intermediate Home, which will take them when they leave here, unless, of course, they have proper homes of their own to go to. Think what the change to a single squalid room, or even to a workhouse infirmary, must be, after years in a bright, pleasant place like this, with delicate food (I hope you don't think us extravagant for giving them that), amusement, and instruction, so far as they are fitted for it, and the most careful and kindest of nursing!"

"In fact, there is a missing link," said Julian, roused to sympathy and interest at once. "And it is a link that is urgently needed, that is evident. Why not start a fund for such a Home at once, and call it the 'Missing Link' Fund?"

"We *have* started a fund," she answered; "but it does not seem to get on much. If only people knew more of our Home, and of the need for such a continuation of our work, I can't but think sufficient help would come."

Julian and Florence looked at each other,—smiled, as they saw in each other's eyes the eagerness to help, then sighed, as they remembered how comparatively small, after all, was the amount that they could do.

AFFLICTED IN MIND AS WELL AS IN BODY.

"May we go over the Home?" said Florence, presently; and the Lady Superintendent gladly led the way to the playroom, to which all the children who could be moved were brought down every day. Some were reading, others playing; but Florence noticed a sadly vacant expression on several little faces, and remarked upon it to the Lady Superintendent, as they left the room.

"Yes," was the rather mournful answer. "Many of our little ones are imbecile, as well as sufferers in other ways. We shall see worse cases upstairs;" and she took them into a room where a little girl was lying who seemed to be quite beyond the reach of human influence, and lay all day listlessly plucking at the bedclothes, her vacant eyes wandering round the room. "I have never been able to attract her attention; she seems quite unaware of our presence," said the Lady Superintendent, sighing; and she led the way to another room, in which were several more little girls.

### IN THE GIRLS' QUARTERS.

Florence was quite relieved to find that two of them were perfectly bright. One, who seemed to have enough toys on the table before her for the stocking of a small shop, amused the visitors by explaining that these were only what she wanted to play with just then—she had put aside all the others. The other, a white-faced little creature, who looked about seven, but was really, she said, fourteen, answered Florence's questions with the self-possession of a woman, and had a funny little superior manner, doubtless the result of a long sojourn with others who must for ever be treated as children In a baby's chair by the fire was a tiny thing, looking as much like an animal as a human being, with hair growing down almost to her eyes, and a strangely-shaped head. "She is really seven, though she looks almost a baby, doesn't she?" said the nurse, who was evidently devoted to her; and when she knelt down by the chair, talking as she might have done to a kitten or a bird, the poor little creature seemed dimly aware that she was being spoken to, and smiled, though without exactly looking at the nurse.

### IN THE BOYS' QUARTERS.

They went on then to the boys' quarters, talked to the boy whose bed little Eben was to have, and looked into a room where two little boys with enormous heads were playing with an air-ball. The Lady Superintendent picked up from the floor a baby boy, with spinal disease, but a very bright little brain, who called himself "Kiddy" (a name which the other boys had given him), and chattered away to her in sweet, merry fashion. With him in her arms, she took the visitors to see a boy who besides being diseased, was deaf, dumb, blind, and imbecile. "I fancy he just hears my voice, and hopes I shall play with him," she said, as he got off his chair, and moved about, evidently in some excitement; and Julian took the poor little fellow under the arms, and began to jump him up and down, to the child's great delight.

### "IF WE CANNOT CURE, WE CAN ALWAYS ALLEVIATE."

"It must be terribly sad for you and the nurses," she said, as the Lady Superintendent led the way downstairs.

"It is; and I find that it is really quite necessary to let our nurses have more change than is given in ordinary hospitals. Still, our work is by no means altogether sad. Just occasionally we do even cure a case; and anyhow, we cannot doubt that our little ones are made far happier than they would be if there were no such home for them to go to. If we cannot cure, we can always alleviate their sufferings; and really a very large part of the suffering of such children comes from want of proper nursing and care. If you saw the difference between a child just brought in, and the same child when it has been here even a month or two, you would not doubt the value of our work."

### THE LADY SUPERINTENDENT'S FANCY.

"Oh, I do not, I do not," said Florence warmly. "I am longing for little Eben to come here, partly because I so much hope that you will know how to bring him out. Compared with some of these poor children he is brightness itself; and I can't help fancying that he knows and understands much more than he is thought to do."

The Lady Superintendent turned to her with a bright look. "Do you know, I often fancy that of all, or nearly all of them," she said. "Those who can speak say such strange, beautiful little things sometimes; and as for those who cannot, we are sure, from their rapt attention and eager looks, that they are sometimes looking at things quite different from those which are actually before them. Who knows but what, being so far removed from this world, they may catch glimpses, at any rate, of another world which is hid from our eyes?"

### EBEN'S LAST HOME.

She dwelt upon this fancy, if fancy it were, more than ever, when little Eben came into the Home. He had not long to wait, after all; for only a week or two after their visit, the Heywoods received a letter to say that all anxiety as to the fate of the lad whose place Eben was to take was over—he had passed away in sleep the night before, and that their little *protégé* could now come as soon as they liked. So Eben went to his last home on earth, and a veritable home it proved to him. His mother was delighted to find that she might call and see him whenever she could spare time; and as for the little fellow himself, though he suffered at times from the terrible abscesses which formed again and again, those last years of his life were, on the whole, happy ones. No question arose as to further provision for him; his little life faded away when he was but eleven years old; and many tears were shed by friends from within and without over the sweet little face, which, with its aureole of soft, curling hair, looked, as Florence remarked, like the face of a sleeping angel.

### LION'S COT.

Not many weeks after his establishment at the Home an inscription was placed over a cot in the Children's Hospital—the scene of Mrs. Heywood's successful experiment—"In loving and grateful memory of our little Lion." And below, his last words: "Lion'll help you."

That done, Julian and his wife were free to turn to other and larger schemes, still in memory of that never-to-be-forgotten little son, whose life had been laid down, as doubtless it would have been spent, in the service of others.

What did they do? What will others do? is the question which should rather be asked. This short history is a mass of facts, not fancies, held together by the slenderest thread of fiction. Descriptions of life among the poor, pictures of hospital wards and their little inmates—all are from real life; and equally real are the needs of those hospitals—needs which have been of necessity but briefly touched on here. Institutions which have for their object the cure or the care of sick and suffering little children need the aid of many who, like Julian and Florence Heywood, will take up the question *con amore*, and make this work—perhaps the most truly Christian in this Christian land of ours—all that it ought to be—all that it might be, if each of us would but lend a hand.

THE END.

